# Pilates based core stability training in ambulant individuals with multiple sclerosis: protocol for a multi-centre randomised controlled trial

**DOI:** 10.1186/1471-2377-12-19

**Published:** 2012-04-05

**Authors:** Jennifer Freeman, Esther Fox, Margaret Gear, Alan Hough

**Affiliations:** 1 Faculty of Health, Education and Society, School of Health Professions, Plymouth University, Peninsula Allied Health Centre, Derriford Road, Plymouth PL6 8BH, UK; 2 Physiotherapy Department, Gilbert Bain Hospital, Lerwick, Shetland ZE1 0TB, UK

**Keywords:** Multiple sclerosis, Mobility, Balance, Exercise, Physiotherapy, Core stability

## Abstract

**Background:**

People with Multiple Sclerosis (MS) frequently experience balance and mobility impairments, including reduced trunk stability. Pilates-based core stability training, which is aimed at improving control of the body's stabilising muscles, is popular as a form of exercise with people with MS and therapists. A replicated single case series study facilitated by the Therapists in MS Group in the United Kingdom (UK) provides preliminary evidence that this approach can improve balance and mobility in ambulant people with MS; further evidence is needed to substantiate these findings to ensure that limited time, energy, finances and resources are used to best effect.

This study builds upon the pilot work undertaken in the case series study by implementing a powered randomised controlled study, with the aims of:

1 Establishing the effectiveness of core stability training

2 Comparing core stability training with standardised physiotherapy exercise

3 Exploring underlying mechanisms of change associated with this intervention

**Methods:**

This is a multi-centre, double blind, block randomised, controlled trial. Eligible participants will be recruited from 4 UK centres. Participants will be randomly allocated to one of three groups: Pilates based core stability training, standardised physiotherapy exercise or contract-relax relaxation sessions (placebo control). All will receive face to face training sessions over a 12 week period; together with a 15 minute daily home programme. All will be assessed by a blinded assessor before training, at the end of the 12 week programme and at 4 week follow-up. The primary outcome measure is the 10 metre timed walk. Secondary outcome measures are the MS walking Scale (MSWS-12), the Functional Reach (forwards and lateral), a 10 point Numerical Rating Scale to determine "Difficulty in carrying a drink when walking", and the Activities-specific Balance Confidence (ABC) Scale. In addition, ultrasound imaging of the abdominal muscles will be performed before and after intervention to assess changes in abdominal musculature at one of the four centres (Plymouth).

**Discussion:**

This pragmatic trial will assess the effect of these exercise programmes on ambulatory people with MS. It may not be possible to extrapolate the conclusions to those who are non-ambulatory.

**Trial registration:**

ClinicalTrials.gov: NCT01414725

## Background

People with MS frequently experience disabling balance and mobility impairments. One component of balance is postural stability of the trunk. This is now commonly termed "core stability" [1]. People with MS have been found to have reduced trunk stability during arm movements in sitting compared to healthy subjects, implying reduced core stability [2].

Pilates based core stability training is a precise, controlled form of exercise using the stabilising muscles of the body [3]. Despite a lack of scientific evidence to support the effectiveness of core stability training in people with MS, it is increasingly being advocated as a treatment strategy. As a consequence core stability training is often integrated into rehabilitation programmes, both on an individual and group basis. Furthermore Pilates based exercise classes are popular with people with MS who pay to participate in these within community leisure settings.

In response to this, the Therapists in Multiple Sclerosis (TiMS) Research Group based in the United Kingdom (UK) [4], undertook a small scale multi-centre series of eight replicated single case studies (ABA design), across five geographically dispersed centres, to explore the effect of Pilates based core stability training on balance, mobility, and balance confidence in ambulant individuals with MS [5]. Visual analysis (trend, level and slope between baseline and intervention phases), together with the 2 standard deviation band statistical analyses showed improvement in at least 6 of the 9 measures for the majority of individuals. Group analyses (repeated measures within-subjects analysis of variance) demonstrated significant improvement between baseline and intervention phases for the 10 metre Timed Walk (p = 0.019), MSWS-12 Scale (p = 0.041), Forward (p = 0.015) and Lateral Functional Reach (p = 0.012). These results provided preliminary scientific evidence to support the use of this intervention in ambulant individuals with MS.

The next stage is to build upon this existing evidence base by improving the methodological limitations of this pilot study. We intend to do this by undertaking an adequately powered multi-centred blinded randomised controlled study, with the *primary aim *of investigating the effectiveness of a 12 week face to face Pilates based individualised core stability training programme. Methodologically sound trials of this nature are essential to move our knowledge forwards in this area.

The design and implementation of an individualised core stability training regimen is believed to require specific skills; both on behalf of the physiotherapist and the patient. Therapists require post graduate training in this skill; and patients need to learn to voluntarily activate muscles (the core stabilisers) that are usually only recruited on an automatic basis [6]. An important *secondary aim*, therefore, is to investigate whether any difference in outcome exists between a Pilates based core stability training approach, and provision of a standardised lower limb exercise programme. Finally, one of the recognised aims of Pilates based-core stability training is to selectively target the deep abdominal muscles in order to optimise the stabilising effect [7]. A *further secondary aim *therefore is to use ultrasound imaging to determine if changes in resting thickness and activation levels of these deep muscles occur following exercise intervention.

## Methods and design

### Design

This will be a multi-centre, double blind, block randomised, controlled trial. There will be two intervention groups and one control group. Ethical Approval has been gained from the National Research Ethics Service, South West 3 Regional Ethics Committee (REC Reference Number: 10/H0106/88), and from the Faculty of Health Ethics Committee at Plymouth University (REC Reference Code: MS/ab). National Health Service (NHS) Research and Development Approval has been gained from the 3 participating NHS Centres.

### Participants

This multi-centre study will involve four UK centres: North Lanarkshire NHS Trust, Glasgow; National Hospital for Neurology and Neurosurgery, University College London Hospitals Trust, London; Newton Abbot Hospital, NHS Devon, Devon; and The School of Health Professions, Plymouth University, Devon.

#### Entry criteria

• Definite diagnosis of MS, according to McDonald's criteria [8]

• Aged 18 years or over

• Able to walk independently with or without use of intermittent or constant unilateral assistance such as walking stick or orthotic brace (4.0 - 6.5 EDSS equivalent)

#### Exclusion criteria

• In relapse or relapse in previous three months [9]

• Any medical condition contra-indicating participation in core stability exercises

• Score < 6 on the Abbreviated Mental Test [10], as an indicator of those whose cognitive difficulties could interfere with the informed consent process, or the ability to fully engage in the exercise programme.

• Current or recent (within past 6 months) participation in core stability exercises

• Current involvement in another interventional research study

### Sample size

The sample size for this study is based on data from the case series study investigating the effectiveness of a Pilates based core stability training programme on balance and mobility in a similar sample of people with MS [1]. Based on this data, 30 people in each group is required to detect a 20% change in score on the primary outcome (the 10 metre timed walk 10MTW), with a power = 0.85 and α = 0.05. A 20% change in the 10MTW has demonstrated to be clinically significant in similar samples [11]. To allow for a 10% drop-out rate 100 participants will be recruited for this study.

### Participant recruitment and group allocation

The Lanarkshire, Newton Abbot and London centres will each recruit 20 participants, and the Plymouth centre will recruit 40 participants.

#### Participants will be recruited via either

i the physiotherapy department at each of the participating NHS centres, or

ii the SWIMS (South West Impact of MS) database in the case of participants living in the Devon region. SWIMS is a population - based database in which longitudinal data is being collected to assess the impact of MS on over 1100 people in Devon and Cornwall, UK. It is run by the Clinical Neurology Research Group, Peninsula Medical School, Universities of Exeter and Plymouth. Potential participants recruited via this avenue will be made aware of this study in two ways; either by an advertisement placed in the twice yearly newsletter which is sent by the SWIMS team to people with MS who are registered on the database; or via a letter of invitation by the Chief Investigator of SWIMS to participants meeting the inclusion criteria. In either case, the study team contact details will be provided to enable interested SWIMS participants to find out more about the study or in taking part. Under the Data Protection Act, the SWIMS team will not pass on any information about SWIMS participants to the core stability study team. It is only once the core stability team have been contacted by the potential participant that they will begin the recruitment and consent process.

Interested participants will be provided with an information pack describing the study; this will be supported by verbal information when requested. Participants will be screened for eligibility by the principal investigators at each site, who will gain written informed consent for those who meet the inclusion criteria.

After consenting to participate and before intervention, participants will be randomised by the principal investigators at each site to one of three groups -intervention A (Pilates based core stability training), intervention B (standardised physiotherapy exercises) or control intervention C (relaxation). A block randomisation procedure (whereby each site is the block) will be used with central concealment through the use of numbered tickets placed in sealed opaque envelopes, organised by the study co-ordinator. The investigators will open the sealed envelopes sequentially and only after the participant's name and other details are written on the appropriate envelope.

### The interventions

All interventions will be delivered by neurological physiotherapists experienced in managing people with MS, and trained in the standard protocols. All will have undertaken formal Pilates training.

#### Intervention A (Pilates based core stability training programme)

This will comprise 12 half-hour individualised face to face training sessions, delivered over 12 weeks, plus an individualised 15 minute daily home exercise programme.

In line with the case series study protocol, the core stability exercises will be selected from a basket of standardised exercises, each with three levels of difficulty appropriate for participants meeting the study's inclusion criteria. This basket of exercises was generated through a consensus process by the case study researchers [4]. Its intention was to reflect current clinical practice. Standardised written instructions and schematic diagrams will be used, a full explanation of which is available at: http://www.therapistsinms.org.uk/downloads/core-stability-exercise-programme-2011-update.pdf

The exercises are designed to challenge trunk control progressively by adding a gradually increasing limb load, and/or reducing the base of support. Stretching will be undertaken prior to or during these exercises to address any mal-alignments. Where necessary, in the first instance, clinicians will facilitate the movements with a "hands on" approach, progressing to a "hands off" approach. Activation of transversus abdominus, in neutral spinal alignment, will be required for each starting position. Exercises will be progressed in response to the abilities of the individual. Each participant will receive a workbook with written and diagrammatic instructions describing their 15-minute daily home exercise programme.

#### Intervention B (standardised exercise programme)

This will comprise 12 half-hour individualised face to face training sessions, delivered over 12 weeks, plus an individualised 15-minute daily home exercise programme. A standardised programme of simple physiotherapy exercises which aims to improve trunk and pelvic stability, lower limb muscle length and strength and balance and control of movement will be used as described by Barrett et al. [12]. This programme is considered reflective of the general exercises typically undertaken within routine clinical practice. In line with Barrett et al's protocol, specific exercises appropriate to each individual will be chosen from a list. They will be progressed where appropriate by choosing more challenging ones from the list at weekly intervals. To mirror the core stability programme, participants in this group will also be instructed to undertake an individualised 15-minute daily home exercise programme.

#### Intervention C - control intervention (relaxation programme)

This will comprise three face to face individualised relaxation sessions (up to a maximum of 60 minutes), provided at 4 weekly intervals, plus a 15-minute daily home programme based around an audio relaxation CD provided to the participants. In an attempt to blind the participant to group allocation, the relaxation sessions utilise contract-relax techniques, wherein the participant concentrates on progressively contracting the muscles prior to relaxing them. Weekly telephone contact/support will also be provided in an attempt to control for attention.

For all groups, people who experience a relapse will stop the intervention programme and will be withdrawn from the study. Should this occur, the site researcher will inform the study co-ordinator/Chief Investigator immediately.

### Assessment

Each participant will be assessed by an independent trained blinded assessor who is unaware of group allocation.

Assessments with the following standardised and validated outcome measures will be undertaken on three separate occasions; at baseline before the intervention (0 weeks), after the intervention (12 weeks) and at follow-up one month after completion of the intervention phase (16 weeks).

#### Demographic and diagnostic baseline data

Demographic (age, gender) and diagnostic (type of MS, number of years since first symptoms, number of years since diagnosis) data will be collected for all participants on a standardised data collection form. In addition details of current medications will be collected, and any changes to these (or other medical interventions) will be noted throughout the course of the study.

#### Outcome measures

Measures will be undertaken in a standardised order to account for practice and fatigue effects. Comfortable shoes and orthoses/walking aids will be worn, which will be the same on each testing occasion. In all timed tests a stop watch accurate to one decimal place will be used.

All of these measures were used in the case series study [5]. All have demonstrated to be easily administered and clinically relevant. All have published data concerning their psychometric properties, demonstrating them to be valid, reliable and responsive in similar MS samples and with similar interventions. This range of measures reflects the differing aspects of mobility and balance that can be affected in MS, which include speed of walking, ability to maintain balance with variations in base of support, confidence with balance, and self-perception of walking ability.

#### Primary clinical outcome measure

1 10 metre timed walk test [13] - this clinician based measure of mobility will be used to assess speed of walking. In line with other studies of ambulant individuals with MS, a single test will be performed at self-selected speed, from a still start.

#### Secondary clinical outcome measures

1 MS 12 item walking scale [14] - this patient based self-report scale of mobility will be used to assess patient perspective on mobility.

2 Functional Reach - forwards and lateral [15].

Functional reach mirrors the everyday activity of reaching for objects beyond arm's length. In line with other MS studies three trials of each test will be performed and the mean determined.

3 Numerical Rating Scale (10 point) to determine "Difficulty in carrying a drink when walking". This was identified as a common functional difficulty by the site therapists in the case series study [1].

4 Activities-specific Balance Confidence (ABC) Scale [16]

This self-report scale rates the persons perceived level of confidence in performing 16 daily activities.

#### Compliance data

Attendance at training sessions will be recorded for all participants. At each training session, therapists will record specifics in relation to the type, level and repetition of exercises undertaken (Interventions A and B), and duration and content (Control Intervention).

All participants will be asked to complete a tick-box diary to record compliance with their home exercise programme. In the case of interventions A and B, this will also include the number of repetitions undertaken.

#### Measures to explore the underlying mechanisms of change

In addition to the clinically based functional outcome measures, in a sub-group of 20 subjects based at Plymouth University, automatic activation of the deep abdominal core stabilising muscles using ultrasound imaging will be measured before (0 weeks) and immediately after completion of the intervention programme (12 weeks). Measurement of abdominal muscle thickness changes during exercise have been shown to be valid measures of muscle activity [17,18]. Measuring both the resting thickness and thickness upon muscle activation in the transversus abdominus and internal oblique muscles will allow us to determine whether differences in the outcomes of the three interventions exist at an impairment level. This will provide important information pertaining to the mechanisms underlying any functional changes which may be observed to occur.

Current literature suggests that 20 hours training are needed to ensure the acquisition and measurement of ultrasound images is reliable for this purpose [19]. Following 20 hours of training, an intra-rater reliability study will be undertaken to establish reliability of ultrasound measurements on repeated testing. A standardised protocol will be used for image acquisition. Images for each of the first 20 consecutive participants at Plymouth University will be taken before and after the intervention period. Participants will be imaged in supine, both at rest and with an active straight leg raise of their strongest leg to automatically activate the deep abdominal muscles [17]. The ultrasound transducer will be placed on the mid axillary line in between the 12^th ^rib and iliac crest on the contralateral side to the leg being lifted. Participants will lie in supine with arms crossed resting on their chest. An image clip will be taken during quiet respiration (image at rest) and then a command will be given to lift the contralateral leg 5 cm at which time a further image clip will be recorded (image of automatic muscle activation). This procedure will be repeated 3 times. The ultrasound image clips will be transferred to a computer and analysed off-line, where the thickness of the transversus abdominus and internal oblique muscles will be measured as a measure of muscle size and activity during both quiet respiration and automatic activation [17].

### Statistical analyses

Statistical analyses will be undertaken using SPSS version 19.0 (SPSS Corp, Chicago, Illinois, USA). Demographic and diagnostic characteristics and baseline data will be summarised by descriptive statistics.

#### Analysis of primary clinical outcomes

The primary analysis will be an intention to treat comparison of the treatment assigned at randomisation. For each of the two primary outcomes, the treatment effect will be tested and presented at the two-tailed 5% level with no adjustment of multiple testing. Treatment effectiveness will be assessed in terms of the difference in change (follow-up minus baseline) for the following comparisons:

• Pilates based intervention versus relaxation (control)

• Standardized exercise programme versus relaxation (control)

• Pilates based intervention versus standardized exercise programme

Ninety five per cent confidence intervals will be produced for the treatment effect on the primary outcomes. Estimates of treatment effects and their standard errors will be tabulated.

#### Analysis of secondary outcomes

Secondary outcomes will be analysed by standard parametric or non-parametric procedures as appropriate. Ninety five per cent confidence intervals will be presented for treatment effects.

The proportion of participants who discontinue with the interventions will be summarised by reason and by intervention group. Results will be reported according to the CONSORT Statement (CONSORT statement 2010) [20].

#### Analysis of ultrasound data

For the sub-group of 20 participants, ninety five per cent confidence intervals will be calculated and presented for resting and automatic activation thickness of transversus abdominus and internal oblique muscles. The effect of type of intervention over time will be tested using analysis of variance at the two-tailed 5% level. We will also undertake a preliminary exploration as to whether factors such as type and duration of diagnosis may impact on physiological state of the core muscles.

## Discussion

Currently there is scant evidence regarding the effectiveness of Pilates based core stability exercise on the balance and mobility of people with MS. It has been suggested that people need to have a level of "body awareness skills" to be able to effectively achieve this voluntary activation of the core stabilisers [6]. This may be particularly difficult for people with MS who can experience a range of sensory, proprioceptive and motor impairments. It is possible that these issues and others (for example the limited face to face contact time that physiotherapists may have with clients) may limit the application of this exercise approach within routine clinical practice. It is also possible that the benefits gained are no greater than that achieved by relatively simple strengthening programmes that load the lower limb, which are frequently provided by therapists of all skill levels within routine clinical practice.

This trial is pragmatic in nature, hence the intention is that the conclusions drawn from the data generated will be directly transferable to clinical practice. Policy makers and therapists implementing physiotherapy programmes will benefit from the results of the trial, by an enhanced evidence base in this area. So too will people with MS who spend time and energy undertaking these exercise regimes, and may choose to self-finance participation in Pilates training programmes. Participants in the trial will only be included if ambulatory (4.0 - 6.5 EDSS) hence it may not be possible to extrapolate the results to less mobile people with MS.

## Conclusions

The results of this clinical trial will show the effect of a 12 week Pilates based core stability programme and a standard physiotherapy exercise programme, in comparison to a placebo control group on subjective and objective measures of balance and mobility in ambulatory people with MS. In addition, the underlying mechanisms of change in the deep abdominal musculature will be evaluated in a sub-set of individuals, with ultrasound imaging.

## Competing interests

The authors declare that they have no competing interests.

## Authors' contributions

JF, MG and AH developed the initial study design and undertook the sample size calculations with advice from the Statistical Department at Plymouth University. EF further developed the study protocol, the randomisation procedure, and the standardised data collection forms; and is responsible for co-ordinating participant recruitment, monitoring randomisation and for project management under the supervision of JF and AH. All authors contributed to writing this manuscript for submission. JF, EF and AH will be involved in compiling and analysing the data. MG will contribute to data interpretation.

## Pre-publication history

The pre-publication history for this paper can be accessed here:

http://www.biomedcentral.com/1471-2377/12/19/prepub
